# A liquid-to-solid phase transition of Cu/Zn superoxide dismutase 1 initiated by oxidation and disease mutation

**DOI:** 10.1016/j.jbc.2022.102857

**Published:** 2022-12-31

**Authors:** Siyu Gu, Ming Xu, Long Chen, Xiangyan Shi, Shi-Zhong Luo

**Affiliations:** 1 Beijing Key Laboratory of Bioprocess, College of Life Science and Technology, Beijing University of Chemical Technology, Beijing, China; 2 Department of Biology, Shenzhen MSU-BIT University, Shenzhen, Guangdong Province, China

**Keywords:** aggregation, neurodegenerative diseases, liquid-liquid phase separation, oxidation, SOD1, ALS, amyotrophic lateral sclerosis, fALS, familial ALS, FBS, fetal bovine serum, FRAP, fluorescence recovery after photobleaching, LLPS, liquid-liquid phase separation, sALS, sporadic ALS, SOD, superoxide dismutase, ThT, thioflavin T

## Abstract

Cu/Zn superoxide dismutase 1 (SOD1) has a high propensity to misfold and form abnormal aggregates when it is subjected to oxidative stress or carries mutations associated with amyotrophic lateral sclerosis. However, the transition from functional soluble SOD1 protein to aggregated SOD1 protein is not completely clear. Here, we propose that liquid-liquid phase separation (LLPS) represents a biophysical process that converts soluble SOD1 into aggregated SOD1. We determined that SOD1 undergoes LLPS *in vitro* and cells under oxidative stress. Abnormal oxidation of SOD1 induces maturation of droplets formed by LLPS, eventually leading to protein aggregation and fibrosis, and involves residues Cys111 and Trp32. Additionally, we found that pathological mutations in SOD1 associated with ALS alter the morphology and material state of the droplets and promote the transformation of SOD1 to solid-like oligomers which are toxic to nerve cells. Furthermore, the fibrous aggregates formed by both pathways have a concentration-dependent toxicity effect on nerve cells. Thus, these combined results strongly indicate that LLPS may play a major role in pathological SOD1 aggregation, contributing to pathogenesis in ALS.

Amyotrophic lateral sclerosis (ALS) is a neurodegenerative disease characterized by the selective degeneration of upper and lower motor neurons. Misfolding and pathological accumulation of superoxide dismutase 1 (SOD1) are common characteristic neuropathological features in both familial ALS (fALS) and sporadic ALS (sALS) cases (1, 2, 3). Misfolding and self-assembly of SOD1 turned into neurotoxic oligomers and aggregates gain toxic function, leading to mitochondrial dysfunction, dysregulation of the ubiquitin-proteasome system, disruption of cytoskeletal elements, and sequestration of essential proteins (4, 5, 6), which demonstrates the pathological importance of SOD1 misfolding and aggregation in ALS.

SOD1 is a homo-dimeric protein (7), which can convert superoxide into hydrogen peroxide and molecular oxygen (8, 9). The SOD1 monomer possesses an eight-stranded antiparallel β-barrel with a hydrophobic inner core (10). The remaining structure components are two loops, the electrostatic loop and the zinc loop, that are considered intrinsically disordered protein regions (11) and are essential for the physiological function and structural stability of SOD1. Meanwhile, each monomer contains a catalytically active copper ion, a zinc ion, and an intramolecular disulfide bond, which confer substantial stability to the native fold of SOD1.

Altering the conformational stability of SOD1 by any intracellular changes can lead to the loss of the physiological function of protein and trigger misfolding. Currently, more than 180 natural mutation sites associated with fALS are known to exist in the SOD1 sequence (12) and are believed to be able to disrupt natural folding and lead to an increased propensity for misfolding and aggregation (13, 14). Recent studies have found that diverse disease-associated SOD1 mutations cause a common structural defect arising from the perturbation of the SOD1 electrostatic loop (15). Moreover, a series of studies have shown that some sALS could be caused by posttranslational modification of WT SOD1, especially oxidation (16). Indeed, oxidized SOD1 may be a causal factor to trigger SOD1 aggregation that increases toxicity (1, 17). Besides, the sALS-linked aberrant oxidatively modified WT SOD1, and fALS-linked SOD1 mutants have similar structural features and the same neurotoxic mechanism (16, 18), which implies WT and mutant SOD1 may share a common aggregation mechanism and a pathogenic pathway in ALS. However, it remains unclear how the functional soluble SOD1 transit to the abnormal pathologically folded insoluble form and further generate the fibrillar accumulation in healthy neurons.

Prior to the formation of stable aggregate species, many aggregation-prone proteins involved in neurodegenerative diseases tend to undergo liquid-liquid phase separation (LLPS) (19, 20, 21). LLPS is a regulated process where bio-macromolecules can autonomously form coexisting high and low concentrated demixing (22). The intracellular condensates formed by LLPS are usually complex and adopt heterogeneous multilayered structures with partially solid-like characters. The solid-like phases can emerge from metastable liquid condensates and further form aggregates-resembled cytoplasmic inclusions, which is relevant for the biological functions and many diseases (20, 23, 24).

Here, we demonstrate that SOD1 forms liquid droplets in a crowded environment through LLPS *in vitro*. In addition, abnormal oxidation of SOD1 induces maturation of droplets formed by LLPS, eventually leading to protein aggregation and fibrosis, and involves residues Cys111 and Trp32. Pathological mutations alter the morphology and material state of the droplets and promote the transformation of SOD1 to solid-like oligomers which are highly toxic to nerve cells. Thus, these findings indicate that LLPS could be a necessary process prior to the SOD1 aggregation. The elucidation of this unique SOD1 property could potentially support the development of novel therapeutics for ALS.

## Results

### Misfolding of SOD1 under oxidative stress in cells and *in vitro*

**Figure 1:**
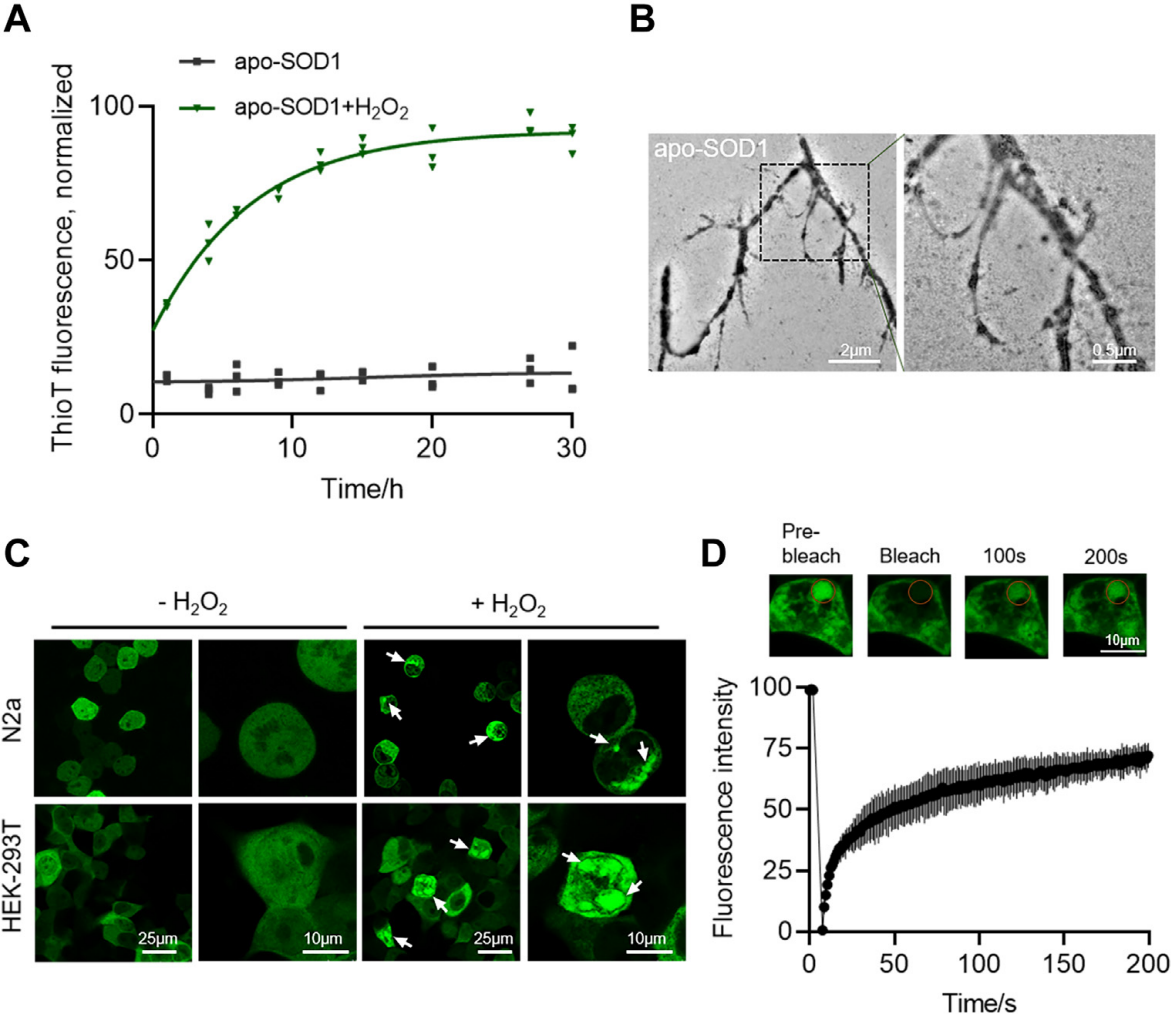
**SOD1 forms misfolding by oxidative stress in cells and *in vitro*.***A*, thio T fluorescence assay of 40 μM apo-SOD1 with or without 200 μM H_2_O_2_. The intensity was normalized with the highest intensity as 100% and the lowest intensity as 0%. n = 3 biologically independent samples, data are presented as mean values ± S.D. *B*, TEM image of 40 μM, oxidized apo-SOD1. Scale bars represent 500 nm. *C*, confocal microscopy images of EGFP-SOD1 aggregates (*arrows*) in both N2a and HEK-293T cells after H_2_O_2_ treatment (100 μM, 3 h). Scale bar represents 25 μm, 10 μm. *D*, FRAP of the condensates formed by oxidized SOD1 in HEK-293T cells. The intensity was normalized with the pre-bleached as 100% and the first time point after bleaching as 0%. n = 3 biologically independent samples, data are presented as mean values ± S.D. Scale bar represents 10 μm. FRAP, fluorescence recovery after photobleaching; SOD, superoxide dismutase; TEM, transmission electron microscope.

Accumulating evidence indicates that SOD1 is predisposed to misfolding and aggregation when oxidized *in vitro* and cells (18, 25, 26). We investigated the physicochemical properties of the oxidized SOD1 aggregation to explore how the dimerization of SOD1 evolved into higher-order oligomers and aggregates. The chelate metal of SOD1 was removed as described (27) to promote fibrillation. *In vitro* aggregation kinetics of metal-deficient SOD1 (apo-SOD1) was measured *via* Thioflavin T (ThT) fluorescence (Fig. 1*A*). Compared with the untreated apo-SOD1, the oxidized form resulted in a significant increase in fluorescence intensity, suggesting the formation of fibrils when treated with H_2_O_2_ *in vitro*. The fibrillization was further confirmed by transmission electron microscope (Fig. 1*B*). In addition, EGFP-tagged SOD1 (EGFP-SOD1) was overexpressed in N2a or HEK-293T cells by transient transfection. It was found that both types of cells formed puncta as shown by the white arrows under oxidative stress (Fig. 1*C*). In contrast, the distribution of EGFP in cells as control was homogeneous in cell cytosol (Fig. S1*A*) under the same conditions. Further, fluorescence recovery after photobleaching (FRAP) experiments revealed that the fluorescence of EGFP-SOD1 puncta recovered within minutes upon photobleaching in HEK-293T cells, suggesting that the contents had high fluidity (Fig. 1*D*). In conclusion, these results demonstrate the occurrence of oxidation can trigger SOD1 fibrillization and induce intracellular inclusions with high mobility.

### SOD1 undergoes LLPS *in vitro*

**Figure 2:**
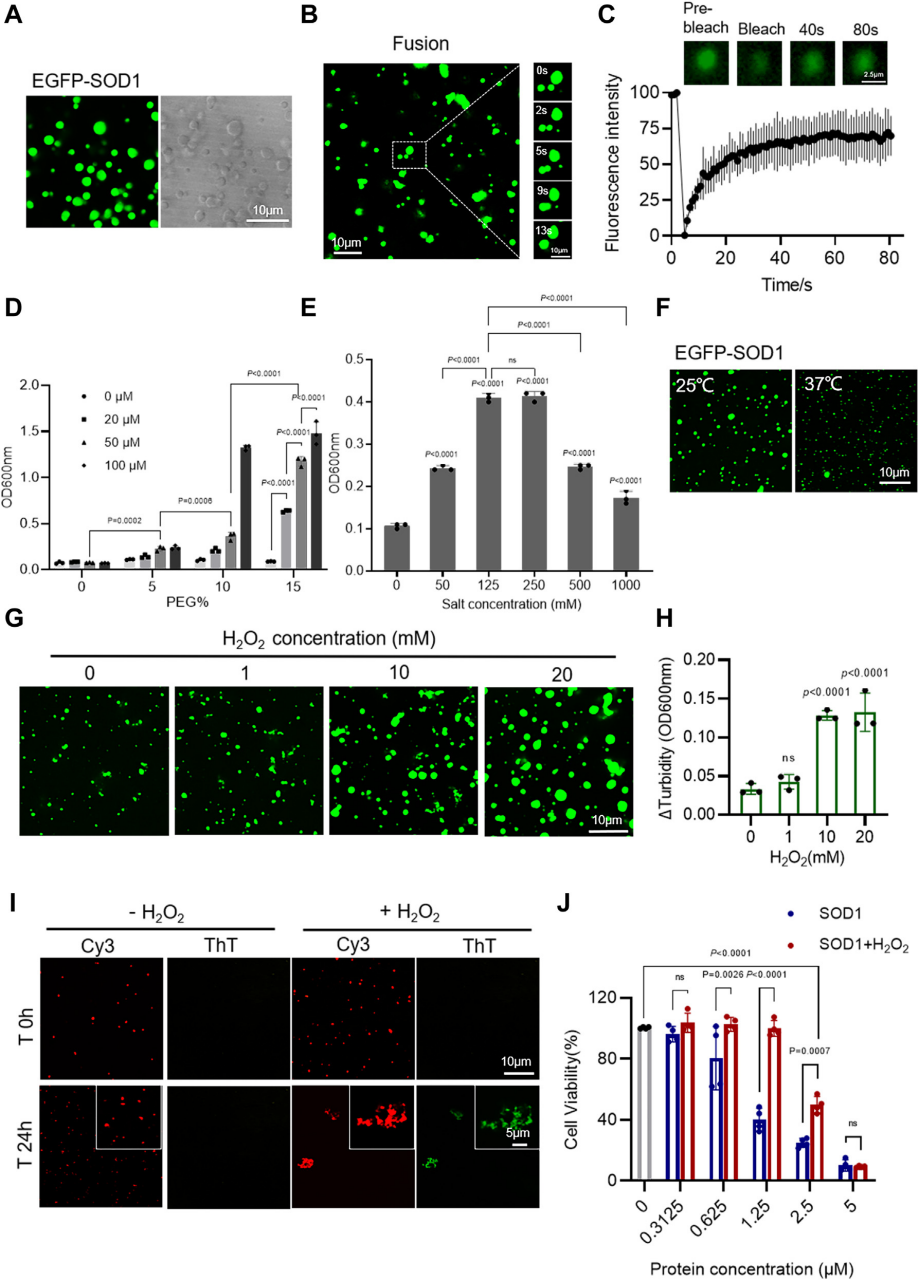
**H**_**2**_**O**_**2**_**promotes SOD1 phase separation.***A*, confocal microscopy images of 100 μM EGFP-SOD1 with 10% PEG. Scale bar represents 10 μm. *B*, droplet fusion experiment of 100 μM EGFP-SOD1 with 10% PEG. Scale bar represents 10 μm. *C*, FRAP of the droplets formed by 40 μM EGFP-SOD1 with 10% PEG. The intensity was normalized with the pre-bleached as 100% and the first time point after bleaching as 0%. n = 3 biologically independent samples, data are presented as mean values ± S.D. Scale bar represents 2.5 μm. *D*, turbidity measurement of 0 to 100 μM EGFP-SOD1 with 0 to 15% PEG. n = 3 biologically independent samples. Data are presented as mean values ± S.D. Data were analyzed by two-way ANOVA with multiple comparisons. *E*, turbidity measurement of 40 μM EGFP-SOD1 with 0 to 1000 mM NaCl concentrations in the presence of 10% PEG. n = 3 biologically independent samples. Data are presented as mean values ± S.D. Data were analyzed by ordinary one-way ANOVA with multiple comparisons. ns, not significant. *F*, confocal microscopy images of 20 μM EGFP-SOD1 at 25 °C or 37 °C in the presence of 15% PEG. Scale bar represents 10 μm. *G*, confocal microscopy images of 40 μM EGFP-SOD1 treated with H_2_O_2_ (0–20 mM, incubated for 2 h at room temperature) in the presence of 136 mM NaCl and 10% PEG. Scale bar represents 10 μm. *H*, difference in turbidity before and after incubation with H_2_O_2_ (ranging from 0–20 mM). Δturbidity = (A600 nm at 2 h − A600 nm at 0 h). n = 3 biologically independent samples. Data are presented as mean values ± S.D. Data were analyzed by ordinary one-way ANOVA with multiple comparisons. ns, not significant. *I*, confocal microscopy images of SOD1(100 μM, 15% PEG) colocalized with 20 μM ThT in the presence or absence of 1 mM H_2_O_2_ (at room temperature for 24 h). Scale bar represents 10 μm. *J*, cytotoxicity of 40 μM SOD1 with or without 200 μM H_2_O_2_ measured by CCK-8 method. Cells were incubated with oxidized or unoxidized SOD1 at 37 °C for 3 days, by which time fibrils had formed in the cells. n = 4 biologically independent samples. Data are presented as mean values ± S.D. Data were analyzed by two-way ANOVA with multiple comparisons. ns, not significant. FRAP, fluorescence recovery after photobleaching; SOD, superoxide dismutase; ThT, thioflavin T.

Proteins that can undergo LLPS usually contain low-complexity intrinsically disordered regions or repetitive multivalent modular domains (28, 29, 30). The high-mobility structural regions of the SOD1 dictate its potential to undergo phase separation prior to aggregation. SOD1 contains the intrinsically disordered and inhomogeneous charge distribution (11, 31) (Fig. S1, *B* and *C*), which may provide sufficient multivalent weak interactions for forming LLPS. To explore this conjecture, EGFP-SOD1 was expressed and purified *in vitro*. Confocal microscopy images of EGFP-SOD1 showed that it forms considerable droplets (Fig. 2*A*). As displayed in Fig. S2*A*, there was a significant difference between the aggregation of EGFP-SOD1 and EGFP. And the droplet fusion experiment also suggested that EGFP-SOD1 droplets could fuse within 13 s (Fig. 2*B*). In addition, both the fluorescent field and bright field of Cy3-labeled SOD1 indicated that SOD1 without EGFP can undergo stable LLPS (Fig. S2*B*). Furthermore, we performed turbidity assay and confocal microscopy experiments with various concentrations of the EGFP-SOD1 and PEG (Figs. 2*D* and S2*D*). The results illustrate that increasing molecular crowding decreases the critical concentration of the protein for phase separation, which suggests that increasing the local concentration *via* molecular crowding is sufficient to induce LLPS of SOD1. Further, FRAP experiments showed that the fluorescence of EGFP-SOD1 and Cy3-labeled SOD1 in the droplets was quickly recovered (Figs. 2*C* and S2*C*) after photobleaching. In summary, the relatively high fluidity and spherical morphology are consistent with SOD1 undergoing LLPS, as opposed to other mechanisms of condensate formation such as gelation or aggregation.

Next, we performed the droplet formation assay with various concentrations of sodium chloride (Figs. 2*E* and S2*F*). Here, we found no evidence for the occurrence of EGFP-SOD1 LLPS in the absence of NaCl. Droplets were most stable at 125 mM NaCl, which is close to the physiological salt concentration. Subsequently, raising the salt concentration led to the dissolution of the condensates (Fig. S2*F*). These results suggest that the LLPS of EGFP-SOD1 is regulated by the salt ion concentration of the solution and is dynamically adjustable. In addition, based on the turbidity and confocal microscopy data plots at different temperatures (Figs. 2*F* and S2*E*), high temperature accelerates the movement of proteins and thus inhibits the LLPS of EGFP-SOD1, which further reflects that the droplets formed by EGFP-SOD1 LLPS can be regulated by temperature. Together, these results suggest that SOD1 LLPS is dynamically tunable and is mediated by electrostatic interactions.

### Oxidation promotes the maturation of SOD1 droplets into fibrous aggregates

As presented above, SOD1 underwent LLPS in a crowded environment *in vitro*. Next, we explored the effect of oxidizing environment on SOD1 LLPS by exposing the observed droplets to various concentrations of hydrogen peroxide (H_2_O_2_). The droplets became significantly larger with the gradual increase of the added H_2_O_2_ concentration (Figs. 2*G* and S2*G*). Compared with that without H_2_O_2_, the solution exhibited enhanced turbidity (Fig. 2*H*). This significantly accelerated LLPS after oxidation may facilitate the interactions and adhesion between droplets, thereby promoting the transition of the droplets to aggregates. The confocal microscope results showed that droplets tended to form clusters of condensates after 4 h of oxidation compared with the case for the unoxidized state (Fig. S2*H*). As shown in Fig. S2*I*, FRAP data indicated that the recovery of the agglomerates formed after oxidation is significantly lower than that of the unoxidized droplets; however, unlike the agglomerates, the droplets still have some mobility.

In addition, unoxidized SOD1 droplets were observed immediately after mixing and were retained after 24 h, while oxidized SOD1 appeared as large irregular aggregates after incubation (Fig. 2*I*). We verified the localization of large irregular aggregates within the cohesive phase, and all aggregates showed intense fluorescence of ThT (Fig. 2*I*). In addition, it has been reported that SOD1 oligomers induce apoptosis of living cells *via* kindling the fibrillization of SOD1 in cells (18, 32). We further focused on the cellular toxicity of the fibrous aggregates formed by oxidation. As seen in Figure 2*J*, significant concentration-dependent cytotoxicity was observed after 3 days of incubation, which was consistent with previous reports (18). Meanwhile, the unoxidized control group was more toxic than the oxidized SOD1, which may be due to the formation of oligomers during the demetallization of SOD1 in the sample preparation process. Oxidation promoted the maturation of droplets to form fibrous aggregates instead of highly toxic oligomers. In fact, oligomers are more toxic than fibrillar aggregates, which is consistent with previous literature (33). In conclusion, these results collectively suggest that oxidation induces the maturation of SOD1 droplets formed by LLPS, and these droplets further developed into fibrous aggregates with concentration-dependent toxicity to neuronal cells.

### Regulation of SOD1 LLPS by oxidation is associated with Cys111 and Trp32

**Figure 3:**
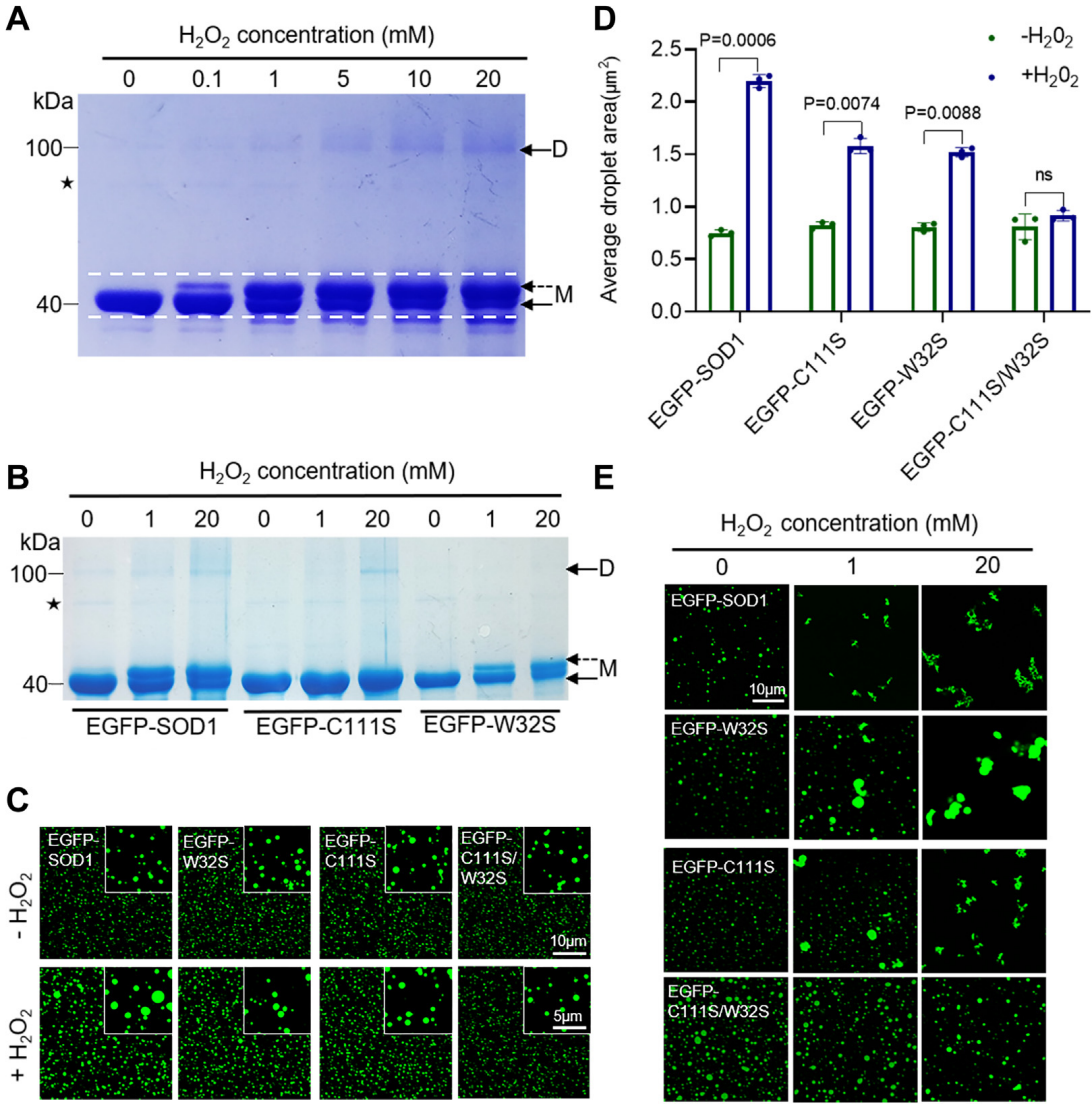
**H**_**2**_**O**_**2**_**regulates SOD1 LLPS associated with Cys111 and Trp32.***A*, SDS-PAGE of 10 μM EGFP-SOD1 was treated with 0 to 20 mM H_2_O_2_ after 2 h at 37 °C. M: monomer, D: dimer. *Dashed arrows* indicated oxidation upshift strips. *Asterisks* indicated impure protein. *B*, SDS-PAGE of 10 μM EGFP-SOD1, C111S, and W32S treated with 0, 1, 20 mM H_2_O_2_ after 2 h at 37 °C. M: monomer, D: dimer. *Dashed arrows* indicated oxidation upshift strips. *Asterisks* indicated impure protein. *C*, confocal microscopy images of 20 μM EGFP-SOD1, EGFP-C111S, EGFP-W32S, EGFP-C111S/W32S with or without H_2_O_2_ (1 mM, 2 h at room temperature) in the presence of 15% PEG. Scale bar represents 10 μm. *D*, area quantification of the droplets from (*B*) *via* intensity thresholding and region of interest (ROI) auto-selection. Histogram of the average area of all droplets. n = 3 biologically independent samples. Data are presented as mean values ± S.D. Data were analyzed by two-way ANOVA with multiple comparisons. ns, not significant. *E*, confocal microscopy images of 40 μM EGFP-SOD1, EGFP-C111S, EGFP-W32S, EGFP-C111S/W32S treated with H_2_O_2_ (0, 1, 20 mM; 10% PEG; 37 °C for 2 h). Scale bar represents 10 μm. LLPS, liquid-liquid phase separation; SOD, superoxide dismutase.

Considerable research effort has been devoted to determining the cause of SOD1 aggregation in response to oxidative damage. Indeed, the two amino acids, C111 and W32 of SOD1, are oxidized *in vivo* and potentially contribute to disease pathogenesis (16, 18, 34, 35). Here, we mutated these two amino acids to generate the C111S, W32S, and C111S/W32S protein variants. We used reduced SDS-PAGE to resolve the EGFP-SOD1 and its variants that were incubated with different concentrations of H_2_O_2_ (Fig. 3*A*). As presented in Figure 3*A*, an additional upper band appeared for the EGFP-SOD1 incubated with >1 mM H_2_O_2_ and migrated more slowly. Besides, as shown by the dashed arrows in Figure 3*A*, the higher concentration of H_2_O_2_ resulted in more obvious upshifted bands, indicating that protein was oxidized (Fig. 3*A*). However, this was not observed when C111S was oxidized under the same conditions for EGFP-SOD1 (Fig. 3*B*), indicating that the upshifted band was due to oxidation of the Cys111, consistent with the previous reports (18, 36). In addition, the same phenomenon was observed for the SDS-PAGE of SOD1 without EGFP tag (Fig. S3*A*), which further confirms our conclusion.

An upshifted band but no dimeric band was observed for the EGFP-W32S sample, which was different from the case for EGFP-SOD1 under the same conditions (Fig. 3*B*). The dimerization of SOD1 presented in the SDS-PAGE under reduced condition indicated that dimers were not formed *via* disulfide bonding, but rather a covalent linkage between monomers through the oxidation of W32 to ditryptophan species.

Next, we explored the contribution of C111 and W32 in the oxidative regulation of SOD1 LLPS. Compared with EGFP-SOD1, EGFP-C111S and EGFP-W32S attenuated the droplet formation enhancement effect introduced by oxidation at room temperature, while EGFP-C111S/W32S completely eliminated it (Fig. 3, *C* and *D*). At 37 °C, EGFP-SOD1 was irregularly aggregated in the presence of H_2_O_2_ (Fig. 3*E*). FRAP assay demonstrated the low mobility of those condensates (Fig. S3). However, both single and double-point mutants mostly remained at the droplet state under 1 mM H_2_O_2_ at 37 °C. When the H_2_O_2_ concertation was higher, similar to EGFP-SOD1, EGFP-C111S and EGFP-W32S could only form large irregular aggregates, while EGFP-C111S/W32S remained as round condensate (Fig. 3*E*). In conclusion, the above results strongly suggest that H_2_O_2_ regulates LLPS by oxidizing the C111 and W32 of SOD1, leading to the transition from droplets to oligomers and further transformation to aggregates.

### Pathological SOD1 mutations alter droplet morphology and accelerate fibrous aggregates formation

**Figure 4:**
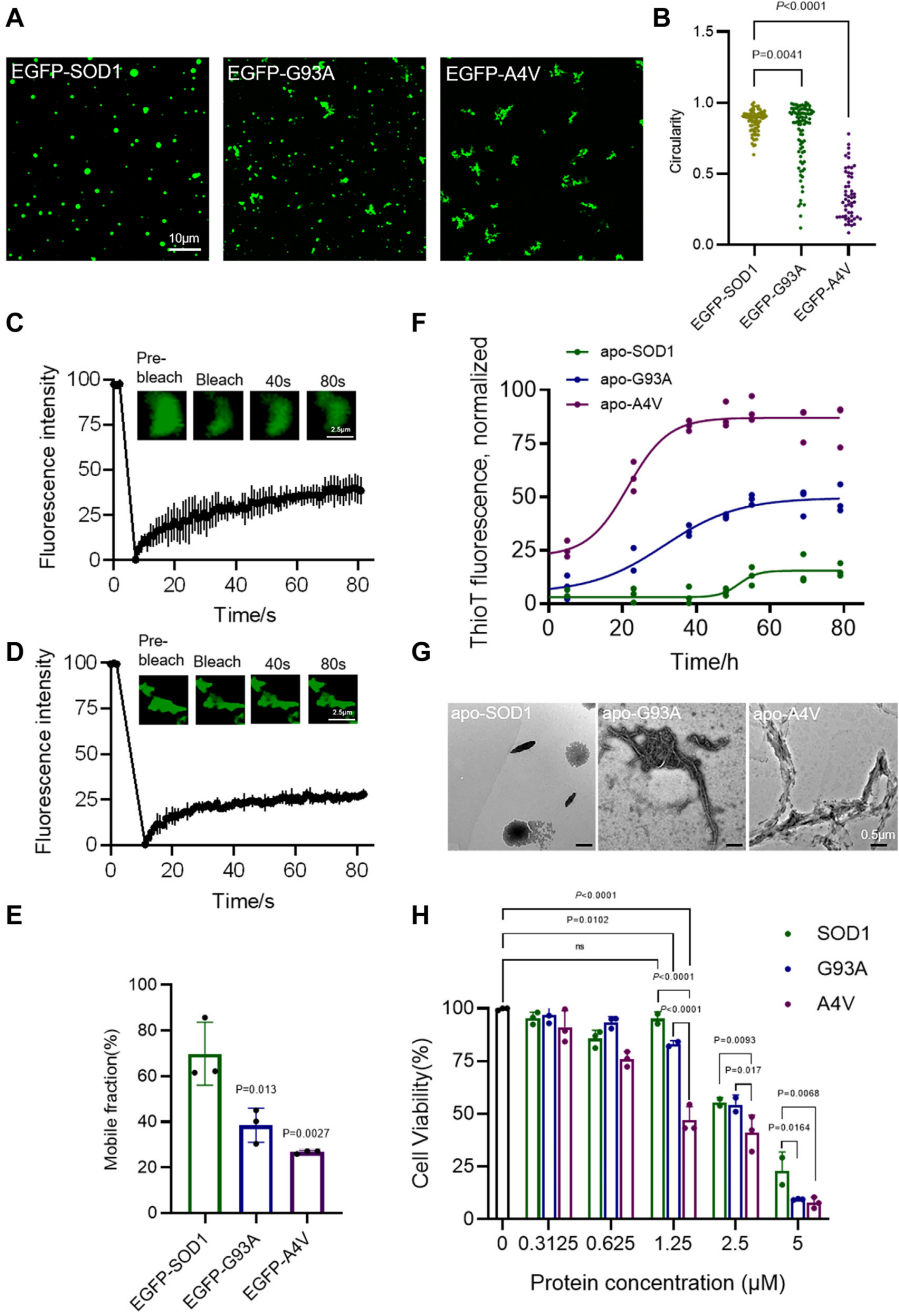
**ALS mutations induce SOD1 protein aggregation.***A*, confocal microscopy images of condensates formed by 50 μM EGFP-SOD1 or mutants with 10% PEG. Scale bar represents 10 μm. *B*, circularity quantification of the droplets/condensates in (*A*) *via* intensity thresholding and region of interest (ROI) auto-selection, circularity = 4π × [Area]/([Perimeter]∧2) ranges from 0 (infinitely elongated polygon) to 1 (positive circle). Dot plot of circularity of all droplets/condensates. n = 3 biologically independent samples, data were analyzed by ordinary one-way ANOVA with multiple comparisons. *C*, FRAP of the condensates formed by EGFP-G93A. The intensity was normalized with the pre-bleached as 100% and the first time point after bleaching as 0%. n = 3 biologically independent samples, data are presented as mean values ± S.D. Scale bar represents 2.5 μm. *D*, FRAP of the condensates formed by EGFP-A4V. The intensity was normalized with the pre-bleached as 100% and the first time point after bleaching as 0%. n = 3 biologically independent samples, data are presented as mean values ± S.D. Scale bar represents 2.5 μm. *E*, quantification of mobile fraction of EGFP-SOD1 or mutants. n = 3 biologically independent samples. Data are presented as mean values ± S.D. Data were analyzed by ordinary one-way ANOVA with multiple comparisons. *F*, ThT fluorescence assay of 40 μM apo-SOD1 and mutants. n = 3 biologically independent samples. Data are presented as mean values ± S.D. *G*, TEM images of 40 μM apo-SOD1 and mutants. Scale bars represent 500 nm. *H*, cytotoxicity of 40 μM SOD1 and mutant aggregates measured by CCK-8 method. Cells were incubated with protein at 37 °C for 3 days. n = 3 biologically independent samples. Data are presented as mean values ± S.D. Data were analyzed by two-way ANOVA with multiple comparisons. ns, not significant. ALS, amyotrophic lateral sclerosis; FRAP, fluorescence recovery after photobleaching; SOD, superoxide dismutase; TEM, transmission electron microscope; ThT, thioflavin T.

Studies have shown that almost all ALS mutations disrupt molecular packing and reduce the likelihood of SOD1 filling the canonical maturation state, which promotes SOD1 aggregation (37, 38, 39). Furthermore, as demonstrated above, SOD1 underwent LLPS prior to oxidation-induced aggregation. To examine the effect of disease-associated mutations on LLPS, two typical fALS-SOD1 mutants (A4V and G93A) were expressed and purified. Confocal images showed that EGFP-G93A and EGFP-A4V formed amorphous droplets or aggregates compared to EGFP-SOD1 (Fig. 4*A*). The mutants also formed irregular aggregate-like entities in the cells (Fig. S4*A*). In addition, the circularity of EGFP-A4V aggregates was lower than 0.5, indicating more intense aggregation (Fig. 4, *A* and *B*). In contrast, droplets formed by nonpathological SOD1 mutations (K75A, T88V, K75A/T88V) exhibited WT-like behavior (Fig. S4*B*). Moreover, FRAP experiments were performed to assess the recovery kinetics of EGFP-G93A and EGFP-A4V condensates. The results elucidated that the fluorescence signals of EGFP-G93A and EGFP-A4V droplet/condensate recovered with a smaller overall mobile fraction of ≈ 38% and 26%, respectively, after photobleaching (Fig. 4, *C* and *D*). Different from the case of EGFP-SOD1, EGFP-G93A and EGFP-A4V condensates behaved more like a gel or solid state based on the slow fluorescence recovery (Fig. 4*E*). We further examined the formation of fibrous aggregates. Compared with the WT (Fig. 2*I*), we found that cohesions of the G93A and A4V variants colocalized with ThT after 24 h (Fig. S4*C*). Under conditions that promote aggregation, G93A and A4V variants were ThT-positive (Fig. 4*F*) and contained fibrillar structures (Fig. 4*G*). The kinetics of aggregation exhibited a lag phase followed by a growth phase and subsequent plateau (Fig. 4*F*). Besides, we observed that the A4V variant had a higher propensity to aggregate than the G93A variant (Fig. 4*F*). There was no significant aggregation of SOD1 in the measurement conditions (Fig. 4, *F* and *G*). Furthermore, consistent with the oxidative results, the fibrous aggregates produced by the mutants exhibited significant concentration-dependent cytotoxicity, with the A4V mutation being more toxic (Fig. 4*H*). At the same time, SOD1 also shows some toxicity, which may be due to the formation of toxic oligomers by demetallization during the preparation of SOD1 samples. However, the SOD1 mutants exhibit much higher toxicity. In conclusion, these results imply that pathological mutations of SOD1 can result in alteration of the LLPS behaviors, reduction of the condensates mobility, and formation of the fibrous aggregations that are toxic to neuronal cells.

## Discussion

The pathological inclusions of SOD1 are closely related to the pathogenesis of ALS disease and are considered to be important diagnostic markers and targets for therapeutic intervention. Moreover, studies have shown that the droplet environment of the protein is very favorable for fibrous aggregates formation (40, 41, 42), and highly enriched phase-separated proteins usually exhibit a greater tendency to aggregate. Our current work demonstrates that SOD1 can assemble into protein-rich droplets *via* LLPS prior to the formation of aggregates.

In mild crowded environment conditions, SOD1 readily undergoes phase separation (Fig. 2*A*) that is tunable by variables that modulate electrostatic forces (Fig. 2*E* and S2*F*). The LLPS of SOD1 is not influenced by the EGFP tag (Fig. S2, *A* and *B*). Indeed, the electrostatic loop of SOD1 is exposed outside the protein structure and is considered highly disordered (11, 43). It is speculated that the increased effective concentration of the protein in the presence of PEG promotes contact between protein dimers, which facilitates the multivalent weak interactions between the electrostatic loops or between the electrostatic loops and electrostatic patches on the protein surface and eventually leading to LLPS. Furthermore, the crystal structure of recombinant human SOD1 clearly shows that the salt bridge D76-K75 and T88-G129 were formed in the interface of ordered five SOD1 dimer lattices (Fig. S4*D*) (44). However, a single (EGFP-K75A and EGFP-T88V) or double-points mutation (EGFP-K75A/T88V) does not affect the LLPS status of SOD1 as shown in Fig. S4*B*. Taken together, the LLPS of SOD1 is driven by the overall electrostatic interaction caused by the positive and negative electrostatic distribution on the surface of the protein structure, and it is not altered by a single or double-points mutation.

Although we did not observe significant phase separation of SOD1 *in vivo*, the appearance of soluble condensates after intracellular oxidation followed the same trend as the droplet enlargement due to oxidation *in vitro* (Figs. 1*C* and 2*G*). This may be caused by the truncated and aggregated SOD1 species with a plethora of conformations coexist, as well as that multiple binding partners of SOD1 (45, 46) change the local concentration of SOD1 available for LLPS *in vivo*. So *in vitro* experiments, we chose a higher protein concentration for a better presentation of the LLPS formation and increased the effective concentration of protein by adding PEG, which lowered the threshold for the occurrence of SOD1 LLPS (Figs. 2*D* and S2*D*).

Although the experiments performed with H_2_O_2_ concentrations are different from *in vivo* steady-state H_2_O_2_ levels, they allow us to simulate the process of SOD1 oxidation *in vitro* and further reveal the effects of oxidation on SOD1. SOD1 is now known to be oxidized at three sites: Cys111, Trp32, and the copper-binding site His (47, 48). There is no evidence for His modifications in the cellular environment effects on ALS, but both Cys111 and Trp32 have implications for disease pathogenesis. Therefore, only two disease-related sites are considered here to investigate the association between SOD1 LLPS and protein aggregation. Our results also reveal that H_2_O_2_ induces oxidative damage through the oxidation active sites, C111 and W32, which enhances intermolecular covalent linkages, thereby promoting LLPS and accelerating protein oligomerization (Fig. 3). Studies have elucidated those excessive oxidative modifications promote the release of SOD1-bound metal ligands, exposure of the hydrophobic surface regions, and the increase of protein disorder (15, 49, 50, 51), which may account for the enhanced *in vitro* phase separation and rapid aggregation of SOD1. Furthermore, W32S and C111S mutants prevent SOD1 aggregation formed by oxidation, and various small molecule drugs 5-Fluorouridine (52, 53), Ebselen (54) have been found to target these sites for treatment. Our data indicate that this protective effect may be obtained by retaining SOD1 in LLPS. Together, our work strongly indicates that phase separation is the first step in the oxidation-induced aggregation of SOD1.

**Figure 5:**
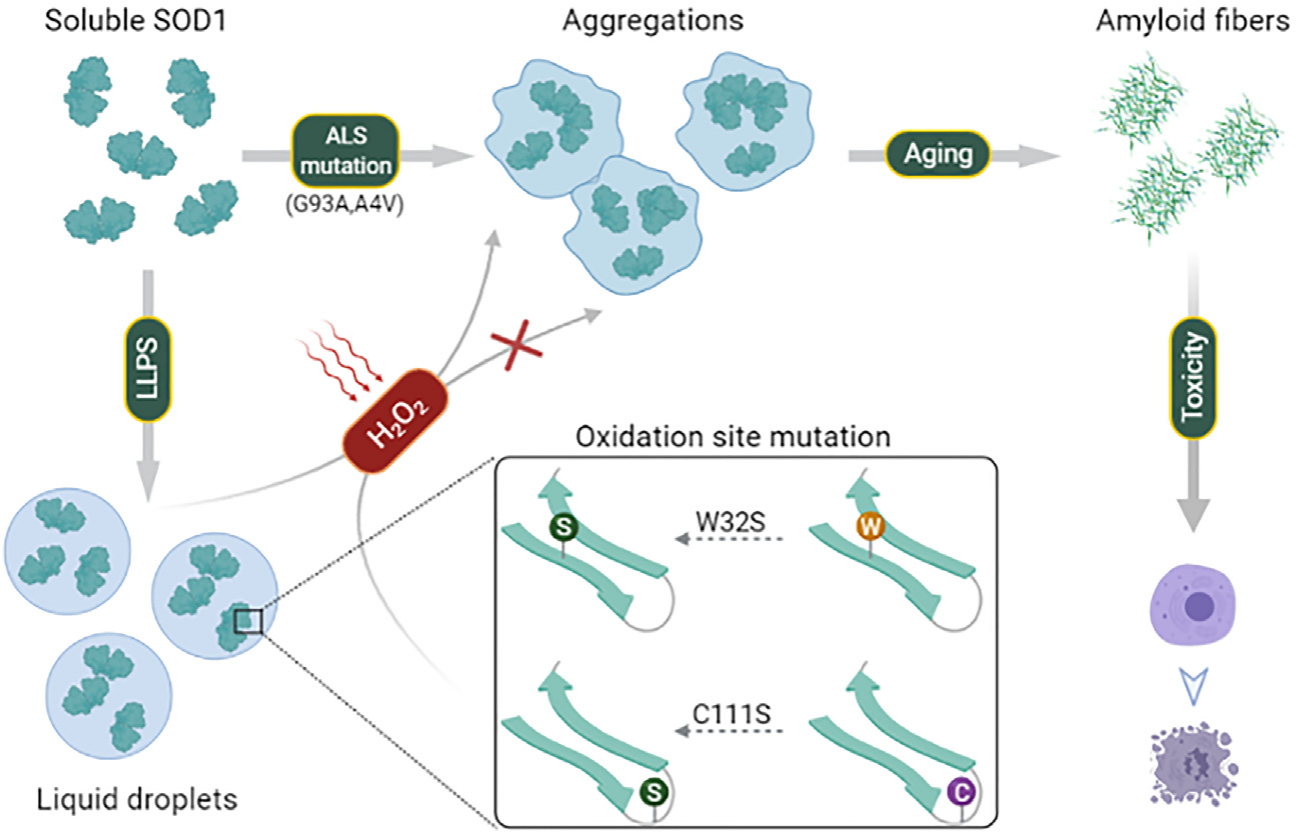
**A model for SOD1 functional LLPS.** Pathological mutations and oxidative damage induce the transition of SOD1 from phase separation to aggregation. LLPS, liquid-liquid phase separation; SOD, superoxide dismutase.

ALS-associated pathological mutations (G93A, A4V) could alter the morphological and physical properties of the droplets, reduce the protein mobility, and promote aggregation (Fig. 4). The A4V mutant is more prone to aggregation than the G93A mutant (Fig. 4, *A* and *F*), which is consistent with the description of the previous results (55, 56). G93 resides at the loop that is distant from metal binding (10), disulphide, and dimer interface regions, while A4 is located at the dimer interface closely related to folding, metallization, and preservation of disulfide bonds (57, 58). The local conformational rearrangement occurs around the A4V mutation site that is close to the dimer interface, promoting the opening of the hydrophobic interface of the SOD1 dimer and the exposure of the hydrophobic interface. These changes lead to the protein being more inclined to aggregate, which likely accounts for the higher degree of aggregation of A4V mutant than G93A mutant. Moreover, unlike oxidation regulation of SOD1 LLPS, pathological mutations directly cause oligomerization of the protein, which has toxic to neuronal cells. It is probably due to more drastic structural changes in the protein caused by the mutation. In addition, aggregates formed by both pathways can further generate fibrous aggregates and have toxicity on neuronal cells (Figs. 2*J* and 4*H*). Thus, our study, for the first time, proposes that SOD1 droplets formation through LLPS is the first step in the polymerization of abnormal SOD1, and the pathogenic aggregation caused by both modalities may be due to a dysregulation of SOD1 phase separation as shown in the model in Figure 5.

In summary, our results clearly demonstrate how SOD1 phase separation, oxidation, and pathological mutations drive the protein undergoing a phase transition from the soluble state to the solid state or forming the fibrous aggregates. The elucidated mechanisms of these processes provide valuable insights into the development of novel therapeutic strategies for the treatment of ALS.

## Experimental procedures

### Disorder and net charge per residue

Disorder of SOD1 was predicted using PONDR-VSL2 (http://www.pondr.com/), and net charge per residue was analyzed using CIDER (http://pappulab.wustl.edu/CIDER/).

### Expression and purification of recombinant SOD1 and mutants

Sequences of all the primers (BGI) used in this study are listed in Table S1. His-SOD1 or His-EGFP-SOD1 genes were constructed in the pET-28a vector (Miao Ling Plasmid). Mutations for SOD1 were created by using the site-directed mutagenesis kit (Beyotime Biotechnology). Recombinant plasmids were transformed into *Escherichia coli* (BL21(DE3), TransGen Biotech) cells for expression. *E. coli* cells were cultured in LB liquid medium at 37 °C until the absorbance (A600) went up to an optimal density (0.8–1.0) and then induced with 0.5 mM IPTG at 16 °C overnight in the presence of copper and zinc as described (59). Recombinant SOD1 and mutants were expressed and purified by the Ni-NTA column (GE Healthcare) in the presence of copper and zinc. The purified proteins were examined by SDS-PAGE gels and stored in elution buffer (50 mM Tris–HCl, 300 mM NaCl, 300 mM Imidazole, pH 7.5–7.8) at −80 °C.

### ThT-binding assays

Apo-SOD1 was demetallized by two-part dialysis (27). Purified SOD1 was first dialyzed in a buffer containing 50 mM NaOAc, 100 mM NaCl, and 10 mM EDTA at pH 4.0 at 4 °C for 16 h. Then, protein was dialyzed in a buffer containing 100 mM Na^+^/phosphate buffer, 100 mM NaCl, 5 mM EDTA at pH 7.4 at 4 °C for 6 h. The apo-SOD1 was filtered through a 0.22 μm membrane. Then, apo-SOD1 was desalted into the buffer (20 mM Tris–HCl, pH 7.4) by ÄKTA pure (General Electric). 40 μM protein with or without 200 μM H_2_O_2_ was incubated for up to 30 h at 37 °C in the presence of 10 μM ThT. SOD1 (WT, G93A, A4V; 40 μM) desalted into the fiber-forming buffer (20 mM Na^+^/phosphate buffer, pH 7.0, 0.1 M NaCl, 5 mM EDTA, 5 mM DTT, and 1% DMF) was incubated with 10 μM ThT for up to 80 h at 37 °C. Fibril formation reactions were set up in a black 96-well plate. ThT fluorescence changes were monitored at 480 nm, with 440 nm excitation, on a SpectraMax M2 (Molecular Devices). The intensity was normalized with the highest intensity as 100% and the lowest intensity as 0%.

### Transmission electron microscope

ThT reaction mixtures (10 μl) were incubated for 2 weeks at 37 °C. Sample preparation and transmission electron microscope analysis were described in detail in a previous study (60).

### Cell culture and transfection assay

N2a cells were cultured in a complete medium containing 44.5% Dulbecco’s modified Eagle’s medium 1 × with glucose (4.5 g/l), 44.5% Minimum Essential Medium Alpha 1 × , 10% fetal bovine serum (FBS), and 1% antibiotics (penicillin/streptomycin). HEK-293T cells were cultured in a complete medium (89% Dulbecco’s modified Eagle’s medium, 10% FBS, and 1% antibiotics). All cells were cultured at 37 °C with 5% CO_2_ in a humidified incubator. Transfection of SOD1 and mutants attached to the pCDNA3.1-EGFP vector (Miao Ling Plasmid) into cells was performed with Lipo8000 transfection reagent (Beyotime Biotechnology). Cell images were captured with a Leica SP8 confocal microscopy with a 100 × objective (oil immersion).

### Phase separation *in vitro*

Purified proteins were desalted into PBS buffer (8 mM Na_2_HPO_4_, 2 mM KH_2_PO_4_, 136 mM NaCl, and 2.6 mM KCl, pH 7.2). For the phase separation of EGFP-SOD1 and mutants, 40 μM of the protein was mixed with 10% (w/v) PEG-8000. The mixed protein solution was immediately loaded into a 96-well plate and imaged within 5 min. For the LLPS oxidation assay, the mixed protein solution (100 μl) mixed with increasing H_2_O_2_ concentrations (0 mM-20 mM) was loaded into a 96-well plate and incubated for the indicated time at the indicated temperature before imaging analysis. For the LLPS colocalized assay, the protein solution mixed with 20 μM ThT was incubated for 24 h at room temperature. Images were captured on a Leica SP8 confocal microscope using a 100 × magnification objective (oil immersion).

### Fluorescence recovery after photobleaching

FRAP assay was conducted using the FRAP module of the Zeiss LSM 780 confocal microscope using 100 × magnification oil objective. A circular region of interest of the condensate was bleached for 6 to 8 s with 100 % laser power of 488-nm or 405-nm lasers (1 AU), respectively and time-lapse images were collected at the rate of 1 s/frame. Signals were normalized with pre-bleached as 100% and 0 s after bleach as 0. GraphPad Prism is used to plot and analyze the FRAP results.

### Turbidity assay

Turbidity (optical density at 600 nm) measurements were conducted in 384-well white polystyrene plate with a clear flat bottom with 40 μl each sample using a SpectraMax M2 (Molecular Devices).

### Labeling of purified SOD1

SOD1 was nonspecifically labeled using Cy3 NHS esters (AAT Bioquest). Mix 7 μl 10 mg/ml of Cy3 monosuccinimidyl ester solution (dissolved in dimethyl sulfoxide) and 7 μl 1 M sodium bicarbonate into a vial containing 100 μl of protein solution with effective shaking at 37 °C for 1 h. Next, sodium bicarbonate was removed by dialyzing the mixed solution into PBS. The stored labeled SOD1 was added to unstained SOD1 in a 10:1 ratio for the experiments.

### Analysis by SDS-PAGE

Protein mixed with various concentrations of H_2_O_2_ was incubated at 37 °C for 2 h in the PBS buffer. After that, N-ethyl maleimide (100 μM final concentration) was used to block the remaining thiols of cysteine residues. Thirty microliters of samples were mixed with 10 μl of 4×SDS loading buffer with β-mercaptoethanol (reducing) respectively, heated for 5 min at 100 °C, and analyzed with SDS-PAGE.

The gels were stained by the staining solution including Kormas Bright Blue G/R-250.

### Cell viability assay

Oxidized SOD1 or ALS-SOD1 mutant fibrous aggregates were formed in their respective aggregation buffers. The cytotoxicity of fibers was determined by Enhanced Cell Counting Kit-8 (Beyotime Biotechnology).

Preparation of protein master mix: 40 μM protein and 200 μM H_2_O_2_ were incubated for about 5 to 6 h to allow oxidation to occur, then the master mix was diluted in a gradient and applied directly to the N2a cells. After that, the N2a cells were cultured in a complete medium without FBS containing various concentrations of the above proteins in 96-well plates for 3 days, by which time fibrils had formed in the cells. Then, 10 μl of CCK-8 was added to each well. Cell viability was determined after 3 h of incubation. The absorbance was measured at 450 nm using SpectraMax M2 microplate reader (Molecular Devices).

### Quantification and statistical analysis

Images were analyzed with Fiji. Statistical analyses were done using the software Graphpad Prism 9.0 (GraphPad Software, Inc).

## Data availability

All data generated or analyzed during this study are included in this article or available from the corresponding author upon request.

## Supporting information

This article contains supporting information (44).

## Conflict of interest

The authors declare that they have no conflicts of interest with the contents of this article.
