# Supplementary material for: A liquid-to-solid phase transition of Cu/Zn superoxide dismutase 1 initiated by oxidation and disease mutation
Source: J Biol Chem. 2022 Dec 31;299(2):102857. doi: 10.1016/j.jbc.2022.102857 (PMC9898760; doi:10.1016/j.jbc.2022.102857)
Supplement: Supplemental Table S1 and Figures S1–S4 [file mmc1.docx]

**A liquid-to-solid phase transition of Cu/Zn superoxide dismutase 1（SOD1）initiated by oxidation and disease mutation**

Siyu Gu^1,‡^, Ming Xu^1,‡^, Long Chen^1^, Xiangyan Shi^2^, Shi-Zhong Luo^1*^

1. Beijing Key Laboratory of Bioprocess, College of Life Science and Technology, Beijing University of Chemical Technology, Beijing, 100029, China

1. Department of Biology, Shenzhen MSU-BIT University, Shenzhen, Guangdong Province, 518172, China

^‡^These authors contributed equally to this work.

^*^ For correspondence: Shi-Zhong Luo, [luosz@mail.buct.edu.cn](mailto:luosz@mail.buct.edu.cn).

**Table S1.** Primers used in this study

| Primer name | Base sequence |
| --- | --- |
| SOD1- BamH1-F | CGGGATCCATGGCGACGAAGGCCGTGTGC |
| SOD1- Xho1-R | CCCCTCGAGTTATTGGGCGATCCCAATTACACCACAAGC |
| C111S-F | CTCTCAGGAGACCATAGCATCATTGGCCGCACAC |
| C111S-R | GTGTGCGGCCAATGATGCTATGGTCTCCTGAGAG |
| W32S-F | GGACCAGTGAAGGTGTCGGGAAGCATTAAAGGAC |
| W32S-R | GTCCTTTAATGCTTCCCGACACCTTCACTGGTCC |
| G93A-F | GACTGCTGACAAAGATGCTGTGGCCGATGTGTC |
| G93A-R | GACACATCGGCCACAGCATCTTTGTCAGCAGTC |
| A4V-F | ATGGCGACGAAGGTCGTGTGCGTGCTG |
| A4V-R | CAGCACGCACACGACCTTCGTCGCCAT |
| K75A-F | GAAAACACGGTGGGCCAGCGGATGAAGAGAGGCATG |
| K75A-R | CATGCCTCTCTTCATCCGCTGGCCCACCGTGTTTTC |
| T88V-F | GAGACTTGGGCAATGTGGTTGCTGACAAAGATGGTG |
| T88V-R | CACCATCTTTGTCAGCAACCACATTGCCCAAGTCTC |


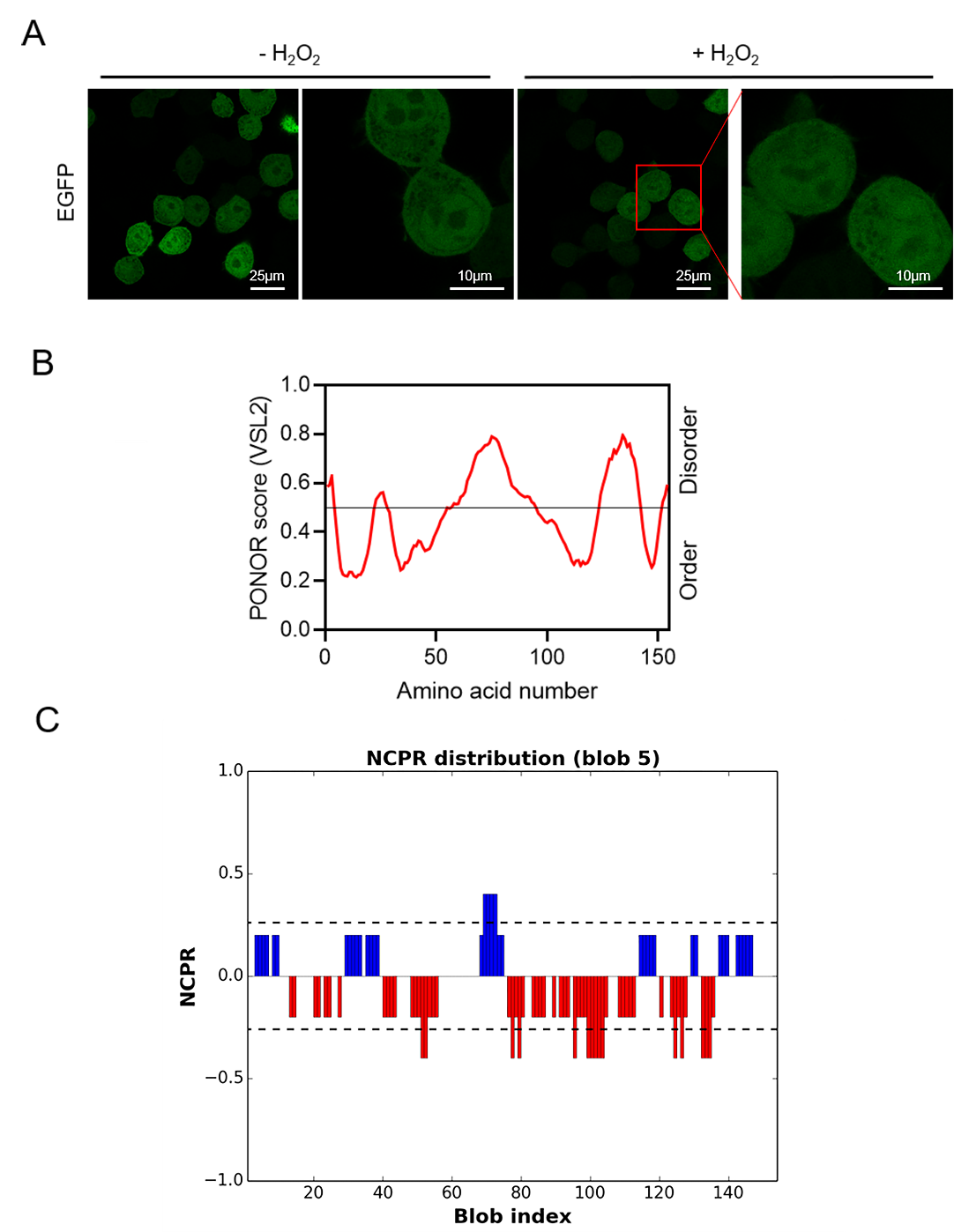


**Figure S1.** **EGFP control in N2a cells and sequence analysis of SOD1**

**A** Confocal microscopy images of EGFP in N2a cells after H_2_O_2_ treatment (100 μM, 3 h). The right-side of the + H_2_O_2_ group is the zoom-magnification image derived from the left image. Scale bar: 25 µm, 10 µm.

**B** Protein sequence and disorder prediction (PONDR-VSL2) of SOD1.

**C** Net charge per residue (NCPR) plot for SOD1 at neutral pH generated using the algorithm available on the CIDER webserver with a five-residue window.


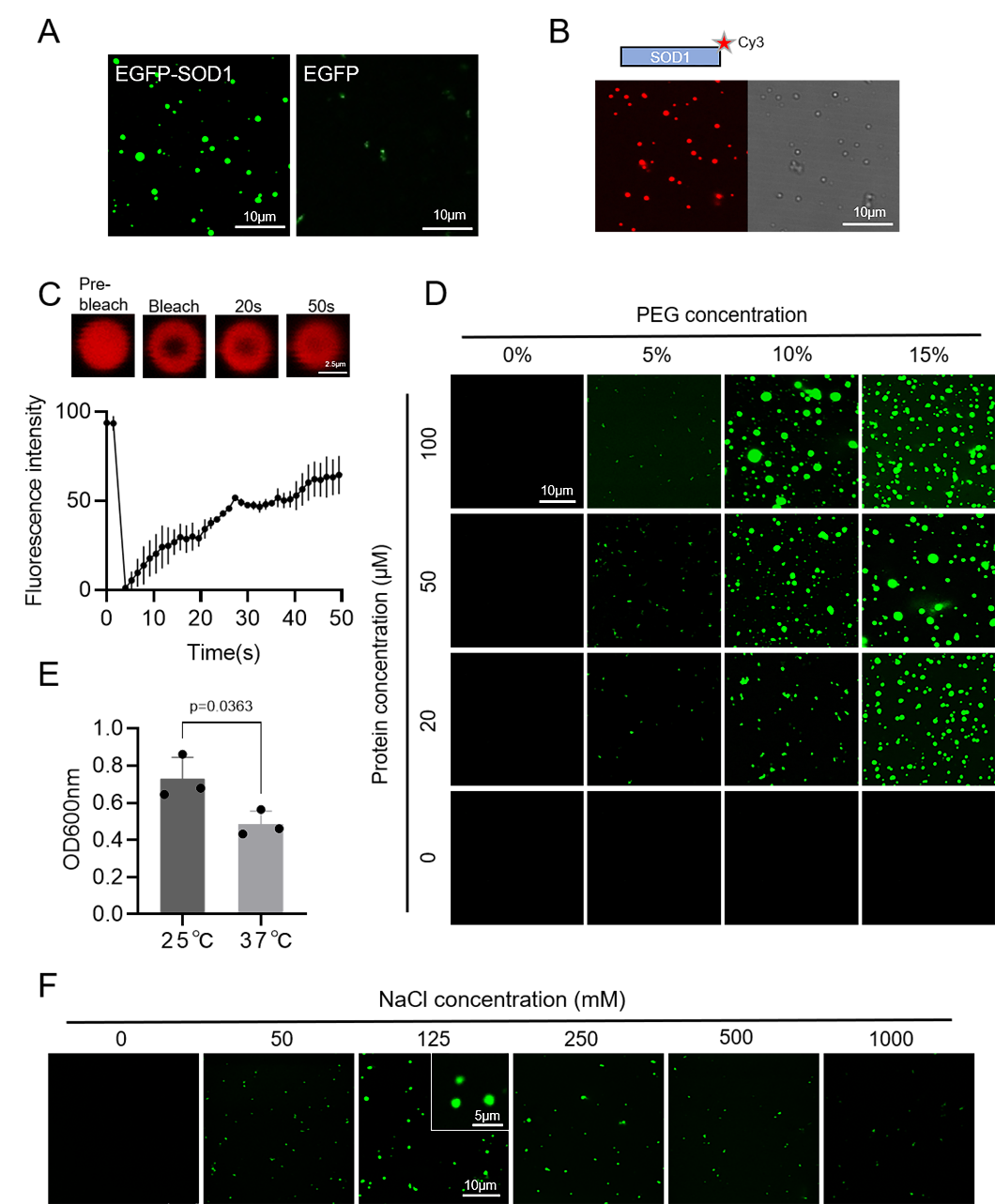


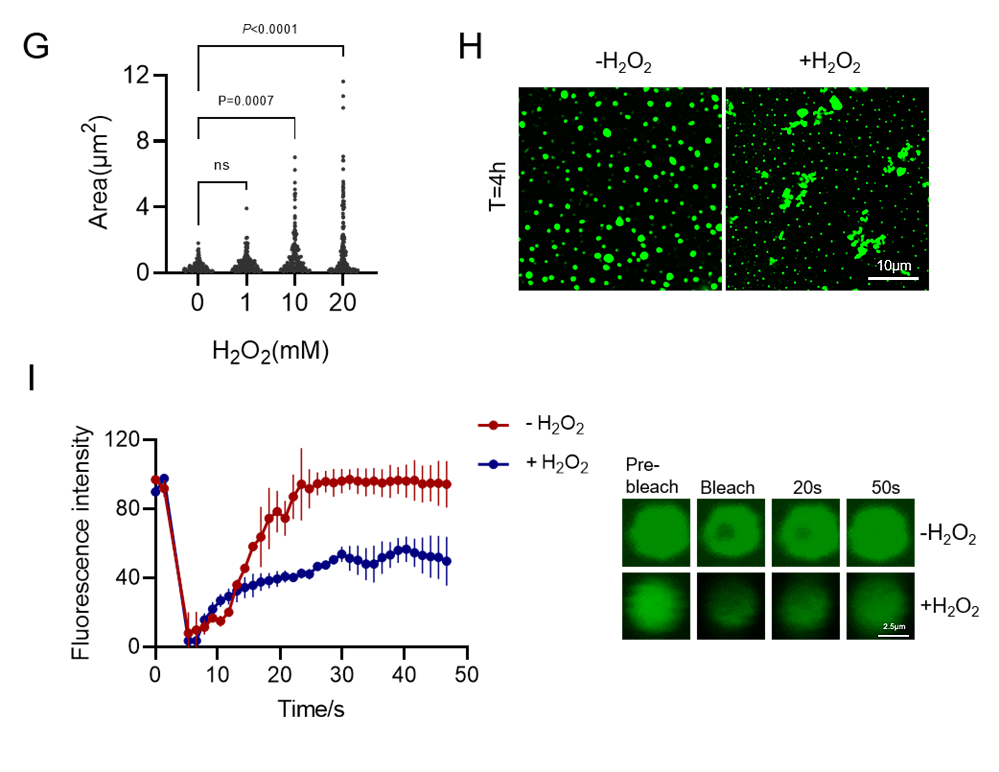


**Figure S2.** **Phase separation of SOD1**

**A** Confocal microscope images of 40 µM EGFP-SOD1 and EGFP with 10% PEG. Scale bar: 10 µm.

**B** Confocal microscopy images of 200 µM Cy3-labeled SOD1 with 10% PEG. Scale bar: 10 µm.

**C** Frap of the droplets formed by 200 µM Cy3-labeled SOD1 with 10% PEG. The intensity was normalized with the pre-beached as 100% and the first-time point after bleaching as 0%. n = 3 biologically independent samples, data are presented as mean values ± S.D. Scale bar: 2.5 µm.

**D** Confocal microscope images of EGFP-SOD1 (0-100 µM) in the presence of PEG ranging from 0-15%. Scale bar: 10 µm.

**E** Turbidity measurement of 20 µM EGFP-SOD1 with 15% PEG at 25℃ or 37℃. n = 3 biologically independent samples. Data are presented as mean values ± S.D. Data were analyzed by Unpaired t test.

**F**  Confocal microscopy images of 40µM EGFP-SOD1 with 0-1000 mM NaCl in the presence of 10% PEG.

**G**  Area quantification of the droplets from (Fig. 2G) via intensity thresholding and region of interest. (ROI) auto-selection. Dot plot of all droplet areas. n = 3 biologically independent samples Data are presented as mean values ± S.D. Data were analyzed by Ordinary One-way ANOVA with Multiple comparisons. ns, not significant.

**H**  Confocal microscopy images of 40 µM EGFP-SOD1 with or without 1mM H_2_O_2_ in the presence of 10% PEG (at room temperature for 4h). Scale bar: 10 µm.

**I**  FRAP of the condensates formed by 40 µM EGFP-SOD1 with or without 1mM H_2_O_2_ in the presence of 10% PEG (at room temperature for 4h). The intensity was normalized with the pre-beached as 100% and the first-time point after bleaching as 0%. n = 3 biologically independent samples, data are presented as mean values ± S.D. Scale bar: 2.5 µm.


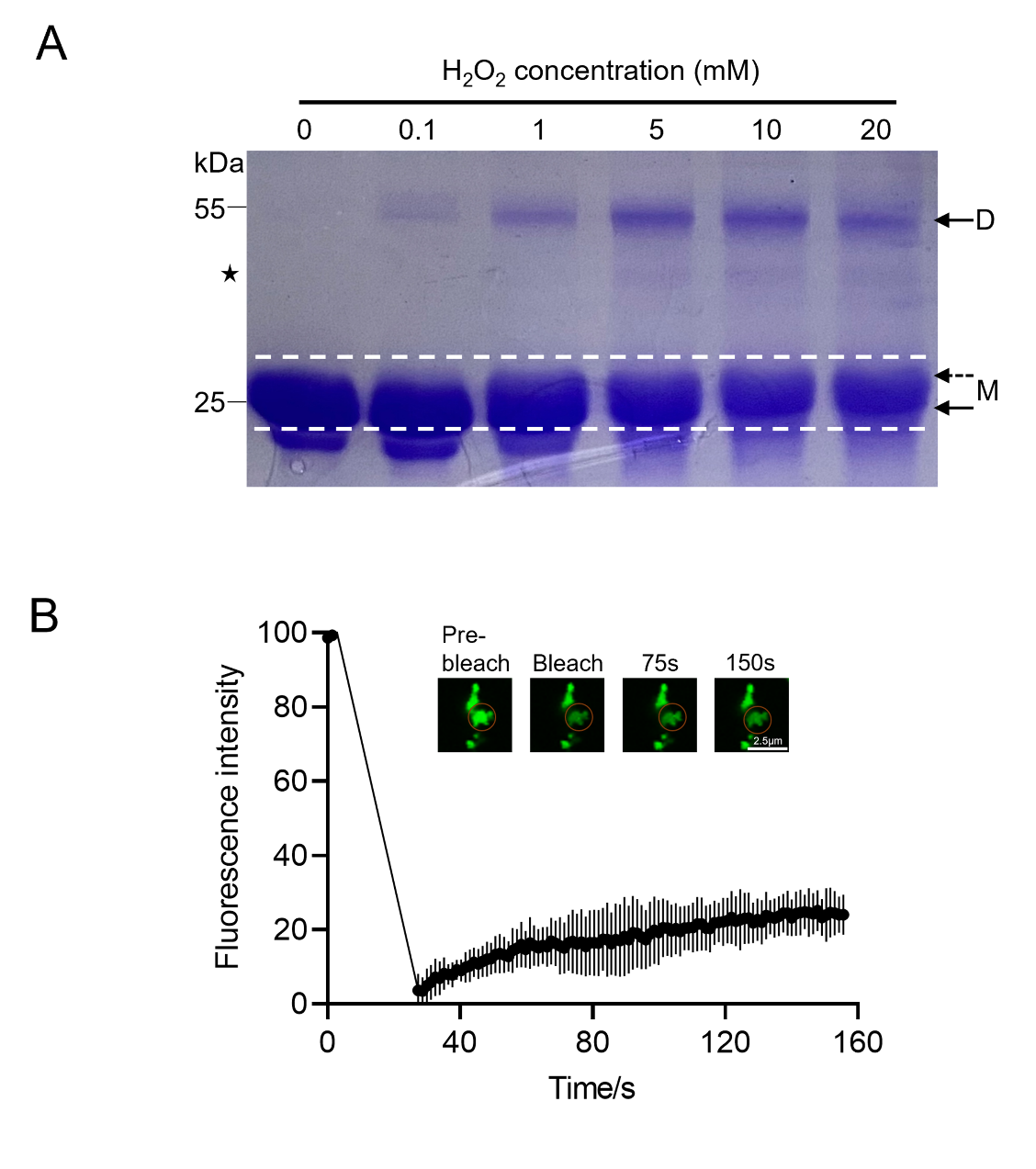


**Figure S3. H_2_O_2_ regulates SOD1 LLPS associated with Cys111 and Trp32.**

**A** SDS-PAGE of 10 µM SOD1 treated with 0-20 mM H_2_O_2_ after 2 hours at 37℃. M: monomer, D: dimer. Dashed arrows indicated oxidation upshift strips. Asterisks indicated impure protein.

**B** FRAP of the aggregates formed by oxidized 40 µM EGFP-SOD1 (20 mM H_2_O_2_, at 37℃ for 2h). n = 3 biologically independent samples, data are presented as mean values ± S.D. Scale bar: 2.5 µm.


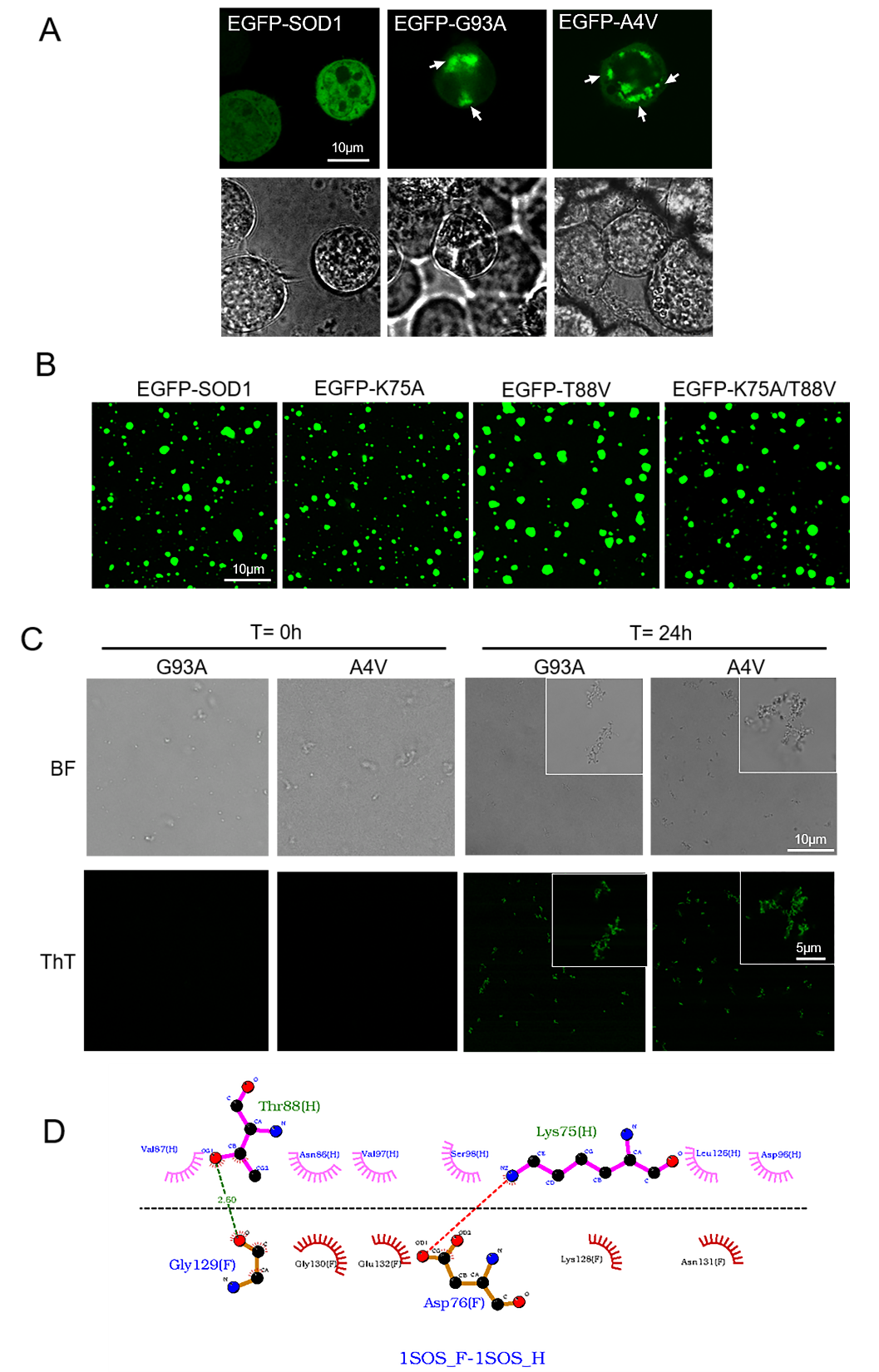


**Figure S4.** **Phase transition and aggregation of SOD1 mutants**

**A** Confocal microscopy images of EGFP-SOD1 wild type and mutants in N2a cells. Scale bar: 10 µm.

**B** Confocal microscopy images of 50 µM EGFP-SOD1 mutants (K75A, T88V, K75A/K88V) with 10% PEG. Scale bar: 10 µm.

**C**  Confocal microscopy images of SOD1-G93A and SOD1-A4V aggregation co-localized with 20 µM ThT. Scale bar: 10 µm.

**D** 2D diagram of the force analysis between two adjacent monomers in five HSOD dimers analyzed with PyMOL Software (PDB:1SOS) (44)
